# Mathematical cognitive structures in Grade 12 students: a mixed-methods concept map study of gender differences and computational ability

**DOI:** 10.3389/fpsyg.2026.1844476

**Published:** 2026-05-15

**Authors:** Zhenping Wang, Junxiong Chen, Jin Liu, Qiaoyu Wu

**Affiliations:** 1 College of Mathematics and Statistics, Henan Normal University, Xinxiang, Henan, China; 2 Faculty of Education, Henan Normal University, Xinxiang, Henan, China

**Keywords:** cognitive structure, computational ability, concept map, gender differences, high school students, mixed-methods research

## Abstract

This study employs a mixed-methods research design, primarily relying on quantitative analysis while incorporating qualitative analysis as a complementary approach. A total of 368 concept maps constructed by Grade 12 students were evaluated using the total proposition scoring method, the Novak’s structural scoring method, and the new structural scoring method. The study aims to explore the characteristics of Grade 12 students’ mathematical cognitive structures and to examine differences across gender and varying levels of mathematical computational ability. This study selected 189 Grade 12 students from four schools in Beijing and Shandong Province, China, focusing on the topics of sequences and trigonometric functions for the concept map drawing test, and after excluding invalid samples, a total of 184 students were included. The study found that Grade 12 students’ mathematical cognitive structures are mainly concentrated between two and five levels. Most students did not form cross-links, and very few exhibited linear structures. Based on the statistical analysis of the frequencies of different concepts and methods appearing in students’ concept maps, significant differences were found in students’ understanding of key concepts and methods in the topics of sequences and trigonometric functions. There were gender differences in students’ mathematical cognitive structure scores (*p* < 0.001), with females scoring slightly higher than males (sequences: Cohen’s *d* = 0.38; trigonometric functions: Cohen’s *d* = 0.19). Students with different levels of mathematical computational ability showed significant differences in their cognitive structure test scores (*p* < 0.001), with the high-ability group > the medium-ability group > the low-ability group.

## 1 Introduction

Cognitive structure is the framework formed by the organization, processing, and storage of knowledge in students’ minds, and learning and problem solving rely on a good cognitive structure (Sweller, 2011; Paas et al., 2003). Relevant research has pointed out that learning outcomes depend not only on the quantity of knowledge but also on how knowledge is organized within the cognitive system and its retrievability (Chi, 2009; Sweller, 2011; Mayer, 2014), and when knowledge is organized and integrated in a meaningful way, learning effects and knowledge retention levels are significantly improved. In other words, as the core carrier of knowledge organization, cognitive structure plays a key role in information retrieval, integration, and transfer, and directly affects learning performance and problem solving ability (Richardson et al., 1996; Schwartz and Bransford, 1998; Bransford et al., 2000; Ericsson, 2006). According to the theory of meaningful learning, existing cognitive structures facilitate the assimilation of new propositions, cultivate connections between concepts, and promote the development and integration of new concepts (Ausubel, 1966; Ausubel, 2012; Gomes et al., 2011). Novak and his colleagues expanded the concept of meaningful learning and developed the concept mapping method to evaluate cognitive structures. Unlike other assessment methods (such as tests and diagnostic interviews), the concept mapping method requires participants to draw concept maps based on their understanding of the structures and connections among concepts stored in their own knowledge base, thereby reflecting characteristics of their cognitive structures (McClure et al., 1999). Therefore, concept maps are considered an effective and convenient assessment tool for detecting cognitive structures (Fenker, 1975; Novak et al., 1983; Evans and Jeong, 2023). In general, when concept maps are used as an assessment method, there are two approaches: qualitative (holistic) and quantitative (Strautmane, 2012; Watson et al., 2016; Ruiz-Primo, 2004; Richmond et al., 2014). The holistic approach is based on general expert judgments regarding structural quality, hierarchy, and form (Hay et al., 2008; Jablokow et al., 2015; Kinchin et al., 2000). The quantitative approach aims to overcome expert subjectivity by using objective indicators (such as the number of concepts, connection density, and hierarchical relationships) (Hay et al., 2008; Anohina and Grundspenkis, 2009).

Mathematics learning is not only related to affective and background factors but also influenced by cognitive factors (Lepore, 2024; Amalina and Vidákovich, 2023). Students with well-developed mathematical cognitive structures can retrieve mathematical knowledge more quickly and flexibly, thereby solving problems (Yang K. et al., 2018; Yang Z. et al., 2018). Existing research has already focused on many elements within mathematical cognitive structures, among which conceptual knowledge and procedural knowledge are not only seen as core components of the development of mathematical ability but also important factors affecting the quality of cognitive structures (Anderson, 1996; Mat Zuki et al., 2024). Conceptual knowledge emphasizes the network of connections between pieces of knowledge, whereas procedural knowledge emphasizes the operational steps and execution of rules for problem solving (Goldman and Hasselbring, 1997; Crooks and Alibali, 2014; Horrocks and Shearman, 2025). Computational ability refers to students’ capacity to perform arithmetic computational accurately and efficiently, apply procedural rules, and complete multi-step calculations in the process of mathematical problem solving. It reflects students’ mastery and application of both conceptual and procedural knowledge (Fuchs et al., 2008; Kikas et al., 2016). Therefore, as an important bridge connecting these two types of knowledge, computational ability plays a key role in the formation and functioning of cognitive structures. Students with higher levels of computational ability are able to retrieve and coordinate relevant knowledge units more efficiently, thereby reducing cognitive load and promoting the integration of conceptual and procedural knowledge within cognitive structures (Sweller, 2011; Paas et al., 2003). Thus, computational ability not only reflects students’ level of knowledge mastery, but also, to some extent, regulates the organization of cognitive structures and their performance in problem solving. In specific research contexts, some researchers have used “concept networks” to conduct node-level analysis of important knowledge in mathematics, transforming the importance of knowledge into quantifiable structural features (Cao and Wu, 2025), thereby providing a quantitative pathway for revealing characteristics of mathematical knowledge structures. Further empirical research analyzing university students’ calculus concept networks found that students with higher cognitive structures have networks with more direct and tighter connections (Yang K. et al., 2018; Yang Z. et al., 2018). Existing research has confirmed that cognitive structure has a positive direct effect on the ability to understand mathematical concepts (Suharto and Widada, 2019). However, these studies have mainly focused on university student populations and primarily examined characteristics of cognitive structures and the effects of concept map interventions in instruction (Evans and Jeong, 2023; Elhilal, 2025). No studies have yet been found that investigate the characteristics of high school students’ mathematical cognitive structures or the differences among different gender groups and different levels of computational ability. In addition, according to Piaget’s theory of cognitive developmental stages, high school students’ cognitive structures are in the stage of development from concrete operations to formal operations, requiring higher levels of abstraction and continuity, and the key to learning lies in forming good cognitive structures (Denervaud et al., 2021; Krieglstein et al., 2022; Lenski et al., 2024). Therefore, it is necessary to conduct research focusing on high school students as a target population. Some researchers have analyzed the organizational characteristics of high school students’ mathematical cognitive structures using network modularity modeling, revealing that high-achieving students demonstrate advantages in knowledge connections and structural organization (Dandan and Zezhong, 2017). Other researchers have employed network data analysis methods, selecting high-performing Grade 10 students as participants and focusing on trigonometric functions as the topic, to examine the organization of mathematical knowledge within well-developed cognitive structures (Yang K. et al., 2018; Yang Z. et al., 2018). In addition, in other disciplines such as chemistry, studies have explored the cognitive structures of students with different levels of achievement (Zhou et al., 2015; Dou et al., 2026). However, existing research in mathematics education has largely focused on describing the overall characteristics of cognitive structures, without further investigating key factors that influence their development, such as group-level variables including gender and mathematical computational ability. Therefore, building on an examination of the characteristics of high school students’ mathematical cognitive structures, this study further explores differences across gender groups and groups with varying levels of computational ability. Moreover, sequences and trigonometric functions are widely regarded as representative and essential topics in high school mathematics, corresponding to the two core domains of “algebra” and “functions” in the secondary mathematics curriculum (National Council of Teachers of Mathematics (NCTM), 2000). These topics encompass both rich conceptual knowledge (e.g., functional relationships and patterns of change) and complex procedural processes (e.g., recursion, identity transformations, and evaluation), thereby effectively reflecting students’ ability to integrate conceptual and procedural knowledge. As such, they are considered both typical and representative (Rittle-Johnson et al., 2001). Therefore, this study selects sequences and trigonometric functions as the content for concept map construction.

Based on this, the present study adopts the total proposition scoring method (Yin et al., 2005), Novak’s classical structural scoring method (Novak and Gowin, 1984), and the new structural scoring method (Tian, 2008) to investigate the following three questions:

*Research question 1*: What are the characteristics of Grade 12 Students’ mathematical cognitive structures?

*Research question 2*: Are there differences in mathematical cognitive structures among Grade 12 Students’ of different genders?

*Research question 3*: Are there differences in mathematical cognitive structures among Grade 12 Students’ with different levels of mathematical computational ability?

## 2 Methods

### 2.1 Participants

In this study, four schools were selected from Beijing and Shandong Province based on differences in school location. One Grade 12 class was randomly chosen from each school as the research sample. The participants were aged between 17 and 18 years. As these students had completed the high school mathematics curriculum, they were considered capable of adequately reflecting high school students’ mathematical cognitive structures. A total of 184 students participated in the survey. Detailed background information of the valid participants is presented in Table 1.

**Table 1:** Demographic information of valid participants.

Background information	School location		School level		Gender	
	Urban schools	County schools	High-performing schools	Average schools	Male	Female
*N*	83	101	83	101	95	89

### 2.2 Procedure

**Figure 1:**
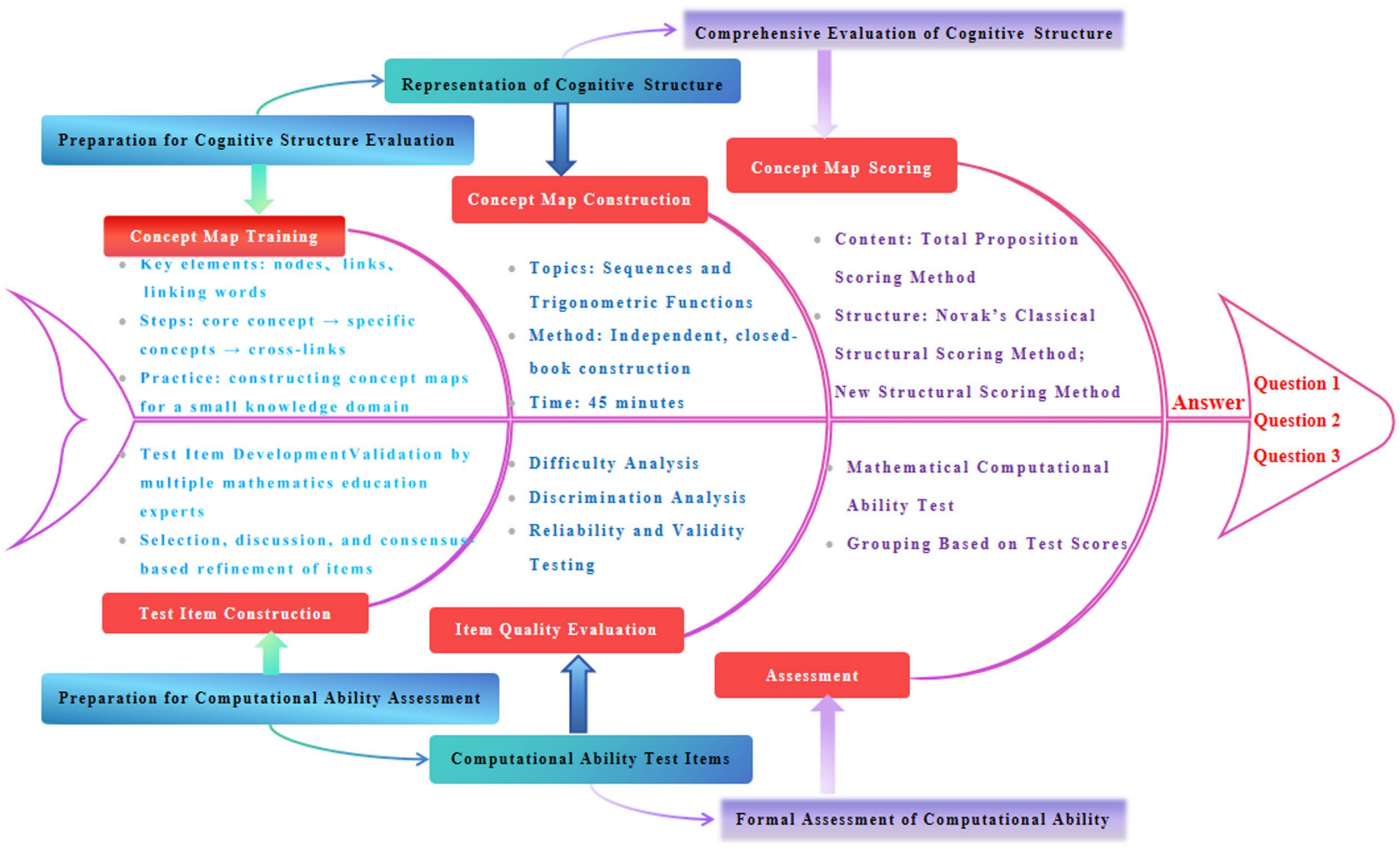
A flowchart structured as a fishbone diagram illustrating the research process for evaluating students’ cognitive and computational abilities. The diagram includes stages such as concept map training, item construction, concept map construction, scoring, and quality evaluation. Arrows connect each stage sequentially, leading to final assessment outcomes addressing three research questions.

The research flowchart is shown in Figure 1. The evaluation of cognitive structures is based on concept maps. We conducted concept map training and practical exercises for students from four classes selected for mathematical cognitive structure research. Following this, students independently created closed-book concept maps for two topics using given high school mathematics content during self-study sessions. The final assessment involved scoring the concept maps through a two-rater evaluation method, where scores were averaged based on evaluations by two independent assessors. For the evaluation of the content dimension of high school students’ mathematical cognitive structure, the total proposition scoring method was adopted(Yin et al., 2005). For the evaluation of the structural dimension of high school students’ mathematical cognitive structure, the Novak’s classical structural scoring method (Novak and Gowin, 1984) and the new structural scoring method (Tian, 2008) were adopted. To examine inter-rater reliability, the scoring results of the two raters under the three methods were analyzed. The Pearson correlation coefficients were 0.889 for the total proposition scoring method, 0.868 for the Novak’s structural scoring method, and 0.857 for the new structural scoring method. All correlations were significant at the 0.01 level, indicating good or above inter-rater reliability across all methods and a high level of scoring consistency. The assessment of computational ability was conducted using a developed set of mathematical operation proficiency test papers. The specific test instrument and detailed quality analysis of the test instrument can be found in the first to sixth pages of the Supplementary materials.

### 2.3 Materials

#### 2.3.1 Scoring methods

The advantages of qualitative analysis are that, even on the basis of incomplete data and information, it can still provide a certain evaluation of teachers’ curriculum knowledge structure and level. However, its disadvantages are that it is greatly influenced by human factors, highly subjective, and it is difficult to ensure that the evaluation results are objective, fair, and precise (Creswell and Plano Clark, 2018; Creswell and Poth, 2018; Merriam and Tisdell, 2016). In contrast, the advantages of quantitative analysis lie in its relatively low susceptibility to human influence, which compensates for the limitations of qualitative analysis, making the evaluation results relatively objective and precise. However, its disadvantage is its strong dependence on data and information; when data are incomplete or insufficient, the evaluation results may deviate significantly from reality (Creswell, 2014). Therefore, this study adopts a mixed-methods approach, with quantitative analysis as the primary method and qualitative analysis as a Supplementary material. Quantitative analysis is mainly used to examine high school students’ mathematical computational ability levels and their cognitive structures of mathematical computation. After collecting students’ concept maps, the evaluators quantitatively processed them using three different concept map scoring methods. Specifically, based on the scoring criteria corresponding to the “Notes” columns in Tables 2–4, the evaluators assigned values to the concepts, linking words, and propositions in students’ concept maps and then summed these values. The resulting scores were used as the basis for assessing the development level of students’ mathematical cognitive structures. Qualitative analysis is mainly applied to the assessment-related components of mathematical computation measurement, including constructing the assessment framework, developing assessment instruments for expert consultation, conducting interviews with teachers, and analyzing students’ typical responses (see Supplementary materials, pp. 7–10).

**Table 2:** Quantitative scoring table for total propositions.

Content indicators	Description	Quantification	
Concepts	Relevance and completeness of concept identification	The number of concepts that meet the criteria described is counted as the score	
Linking words	Effectiveness of the use of linking words	The number of valid linking words is counted as the score	
Propositions	A proposition formed by two concept nodes and a linking word reflects the accuracy of the learner’s understanding (including propositions formed by cross-links)	Vague or incorrect expression	0
		Simple or incomplete expression	1
		Accurate and meaningful expression	2

**Table 3:** Quantitative scoring table for Novak’s classical structural method.

Structural indicators	Description	Quantification scoring
Propositions	Whether the meaningful relationships between two concepts are indicated by linking lines and linking words, and whether these relationships truly exist	Each meaningful and valid proposition is awarded 1 point
Hierarchical levels	The hierarchy in a concept map reflects the learner’s level of knowledge classification; more levels indicate deeper understanding	Each valid hierarchical level is awarded 5 points
Cross-links	The number of cross-links (horizontal connections) reflects the learner’s integrated understanding of the domain knowledge	Each valid and significant cross-link is awarded 10 points; each valid but less integrative cross-link is awarded 2 points
Examples	Instances provided as examples of concepts	Each correct example is awarded 1 point

**Table 4:** Quantitative scoring table for the new structural method.

Category		Description
Spoke structure	First-level spoke structure	Each branch is awarded 1 point; sum the points and multiply by 1.
	Second-level spoke structure	Each branch is awarded 1 point; sum the points and multiply by 2, then sum again.
	……	……
	The total score of all spoke structures in the entire concept map is summed.	
Linear structure		Each concept is awarded 1 point; sum all concepts, then multiply by the hierarchical level of the starting concept in the linear structure.
Line nodes		Each line node is awarded 1 point; sum the total.
Leaf nodes		Each leaf node is awarded 1 point; sum the total.

To evaluate the content dimension of Grade 12 students’ mathematical cognitive structures, the total proposition scoring method was adopted (Yin et al., 2005). The total proposition scoring method focuses solely on propositions and does not take structural organization into account. A proposition is the simplest structural unit in a concept map. Accordingly, this method imposes minimal working memory demands on the rater when analyzing concept maps. Specifically, the rater only needs to sequentially examine the concepts, linking words, and arrows in order to make an accurate judgment. Specifically, before evaluation,the raters first conducted a qualitative analysis of the content quality of the concept maps produced by the students, based on the criteria specified in the “Notes” column of Table 2. They then assigned values according to the number of concepts, linking words, and propositions in the students’ mathematical concept maps. Finally, the total score was calculated to represent the content dimension of students’ mathematical cognitive structures.

Since concept maps can effectively represent knowledge structures, it is also necessary to evaluate this structure accordingly. Therefore, structural scoring places emphasis on the overall configuration of the concept map, with propositions playing a relatively secondary role. The rater is required to interpret students’ knowledge structures based on their own knowledge framework as well as the structure of the standard reference map. For the quantification of the structural indicators of high school students’ mathematical cognitive structure, two evaluation methods were employed.

The first method employed is the Novak’s structural scoring method. Novak proposed a structural scoring system for concept maps, which includes four secondary indicator components and provides specific scoring procedures (Novak and Gowin, 1984). The quantitative operationalization of the concept map structural evaluation indicators is shown in Table 3.

The second method adopts the new structural scoring method (Tian, 2008). This method primarily focuses on scoring the structure of concept maps by summing the scores of four elements. The calculation of leaf nodes is based on the total number of nodes appearing in the concept map. The summary of the quantitative scoring system for the new structural scoring method is presented in Table 4.

#### 2.3.2 Case study of cognitive structure scoring

**Figure 2:**
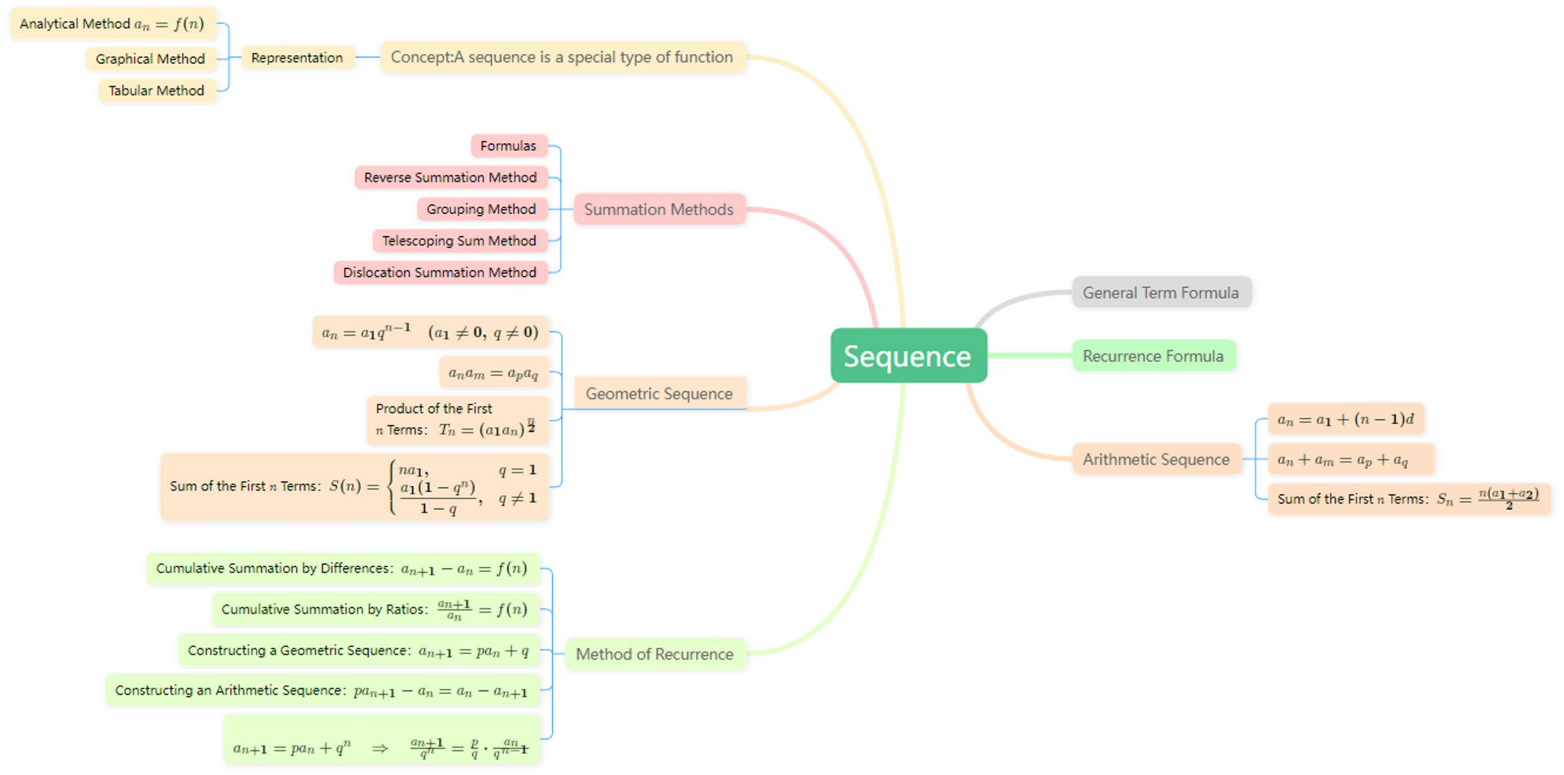
A concept map titled “sequences” illustrating key concepts related to mathematical sequences. The map includes branches for representation methods, summation methods, arithmetic and geometric sequence formulas, and recurrence relations. Nodes are connected by labeled links, and each branch includes relevant formulas and brief explanations to illustrate conceptual relationships.

An example of the concept map is presented in Figure 2. The concept maps drawn by students were scored and analyzed using three scoring methods. More original data images and detailed scoring examples can be found in Supplementary Tables 4–6.

The final score obtained by the total proposition scoring method was 51 points. The concept map drawn by this student contained 33 concepts, indicating that the student had a good grasp of the basic concepts of the “sequence” topic. As shown in Figure 2, the student was very familiar with the recursive methods of sequences and could basically express the knowledge structure contained in “sequences.”The student could provide some examples to explain relevant concepts, for instance: summarizing five different recursive methods for sequences; writing different expressions for arithmetic sequences and geometric sequences, as well as their formulas for the sum of the first n terms. The student scored 12 points on propositions, with some instances of vague or incorrect proposition statements, or statements that were inaccurate or incomplete. Linking words can reflect the vertical level of students’ understanding of knowledge. In Figure 2, the number of linking words used by the student was not many, allowing for a simple hierarchical division of basic concepts, but the deeper-level concepts contained within a concept were not fully or clearly grasped.

The student Novak’s Classic Structure Assessment yielded a score of 52. While demonstrating a well-structured conceptual diagram with multiple hierarchical levels and numerous examples, the absence of cross-linking indicates strong comprehension of sequence-related propositions and conceptual relationships. However, deficiencies remain in understanding the structural connections between concepts and propositions, as evidenced by lower scores in cross-linking components. The lack of horizontal conceptual integration and limited ability to apply knowledge comprehensively further highlight areas for improvement.

The student obtained a score of 83 using the new structural scoring method. From the number of categories within the spoke-like structures, it can be observed that the student has generally mastered the knowledge related to the “sequences” topic and is able to roughly classify concepts at different hierarchical levels; however, the number of third-level spoke-like structures is relatively limited. In terms of the application of formulas, the student is able to describe them through examples and variations, and can correctly state that “a sequence is a special type of function.” The student also provides basic descriptions of the general term formula of sequences and the methods for solving them, and is capable of classifying sequences based on different types and solution methods. Overall, the student demonstrates a relatively comprehensive understanding of sequence-related knowledge and is able to accurately grasp the concepts, representations, general term formulas, and sum formulas of arithmetic and geometric sequences. However, the diagram does not reflect a differentiated understanding of knowledge, indicating that the depth of understanding remains at a relatively superficial level. From the perspective of the spoke-like structures and the number of leaf nodes, the hierarchical organization among different knowledge points is relatively reasonable. The student is also able to integrate and describe knowledge clearly based on their own understanding, forming a relatively well-organized knowledge structure. This suggests that the student has a good level of conceptual understanding in regular learning and engages in reflection and summarization. Nevertheless, the related knowledge appears somewhat fragmented and has not yet formed a systematic structure. Therefore, the level of networked structuring of concepts within the “sequences” topic still requires further improvement.

## 3 Results

Before conducting the statistical analysis, the distributional characteristics of the data were examined using SPSS 27.0. The results showed that the absolute values of skewness for all variables were less than 2, and the absolute values of kurtosis were less than 7, indicating that the data approximately followed a normal distribution. Therefore, independent-samples *t*-tests were used for gender comparisons, and one-way ANOVA was employed to analyze differences among groups with different levels of mathematical computational ability, followed by *post hoc* multiple comparisons. In addition, Cohen’s *d* effect size was calculated to further assess the practical significance of the differences between groups.

### 3.1 Content dimension

Regarding the content dimension of Grade 12 students’ mathematical cognitive structures, the focus was mainly on nodes, connections, and the propositions they form. The nodes provided by students appeared in various forms, including mathematical concepts, formulas, rules, geometric figures, summarized methods, and examples. When evaluating the content of cognitive structures, particular attention was given to the correctness and quantity of the nodes.

#### 3.1.1 Overall performance of the content dimension

This study evaluated the content of mathematical cognitive structures of Grade 12 students in mathematics using three concept map scoring methods for the topics of sequences and trigonometric functions. From Table 5, it can be seen that the overall scores of different students in specific content topics varied greatly. From the perspective of different genders, in both content topics females scored higher than males in the content of cognitive structures, and further analysis using independent samples *t*-tests showed that for the sequence topic, *t* = −3.39 (*p* < 0.001), and for the trigonometric functions topic, *t* = −3.56 (*p* < 0.001), indicating that there were significant differences between male and female students in cognitive structure content scores. This shows that females had significantly higher cognitive structure content scores than males in specific topics. To further measure the practical effect of the differences, Cohen’s d was calculated, with Cohen’s *d* = 0.38 in the sequence topic and Cohen’s *d* = 0.19 in the trigonometric functions topic, and the effect sizes were both relatively small.

**Table 5:** Content scores of Grade 12 students’ mathematical cognitive structures.

Content theme	Group	*N*	Mean	SD
Sequences	Total	184	33.38	18.19
Trigonometric functions	Total	184	36.56	20.97
Sequences	Male	95	28.13	18.53
Sequences	Female	89	35.16	18.07
Trigonometric functions	Male	95	34.67	21.31
Trigonometric functions	Female	89	38.71	20.65

#### 3.1.2 Specific performance of the content dimension

For the sequences functions, students’ cognitive structure content was analyzed, and the main concepts and methods were summarized (see Supplementary Tables 7, 8). The analysis reveals the following characteristics regarding students’ understanding of basic concepts in sequences: on one hand, there are obvious individual differences in the number of basic concepts presented by students, with the highest number reaching 40. On the other hand, frequently used concepts tend to be broader in scope, such as the definitions of arithmetic and geometric sequences, the sum of the first n terms, and the general term formulas of arithmetic and geometric sequences. These concepts appear frequently in students’ concept maps, whereas less commonly used, narrower concepts appear less frequently. Regarding the main methods for solving sequence problems, students’ cognitive structures include 11 types of methods, each appearing with a frequency above 15% in the concept maps. Among these, the method with the highest frequency is the “misalignment subtraction method” (53.26%), while the lowest frequency methods are the “cumulative multiplication method” and “sequence construction method” (15.76%).

For the trigonometric functions, students’ cognitive structure content was similarly analyzed, and the main concepts and methods were summarized (see Supplementary Tables 9, 10). Regarding basic concepts, there were also significant individual differences, with the maximum number of concepts reaching 36. Frequently used core concepts of trigonometric functions appeared more often in concept maps, whereas less commonly used concepts appeared less frequently. Concerning the main formulas and methods for solving trigonometric function problems, students’ cognitive structures included 10 primary methods, each appearing with a frequency above 15%. The most frequently used formula or method is the induction formula (72.83%), while the least frequently used is the special value method (15.21%).

### 3.2 Structural dimension

The structural aspect of Grade 12 students’ mathematical cognitive structures was evaluated using structural scoring methods, which emphasize the arrangement of the concept map while placing propositions in a relatively secondary position. Two scoring methods were used for structural analysis: the first is Novak’s classical structural scoring method, and the second is the new structural scoring method (Table 6).

**Table 6:** Structural scores of Grade 12 students’ mathematical cognitive structures.

Scoring method	Content topic	Group	Mean	Standard deviation
Novak’s classical structural scoring	Sequences	Overall	47.78	15.32
		Male	46.87	17.53
		Female	48.39	14.07
	Trigonometric functions	Overall	39.28	13.32
		Male	40.07	16.53
		Female	38.89	12.07
New structural scoring method	Sequences	Overall	67.31	25.32
		Male	68.25	27.14
		Female	66.13	24.71
	Trigonometric functions	Overall	46.35	18.42
		Male	45.57	20.17
		Female	46.63	19.71

#### 3.2.1 Overall performance of the structural dimension

According to the Novak’s classical structural method, students’ mean structural score for the sequences topic was 47.78 with a standard deviation of 15.42, and females scored higher than males. Further analysis using an independent samples *t*-test showed that *t* = −0.76 and *p* = 0.54 > 0.05, indicating no significant difference in scores between male and female students. According to the new structural method, students’ mean structural score for the sequences topic was 67.31 with a standard deviation of 25.32, and males scored higher than females. Further analysis using an independent samples *t*-test showed that *t* = 0.58 and *p* = 0.62 > 0.05, indicating no significant difference in scores between male and female students.

According to the Novak’s classical structural method, students’ mean structural score for the trigonometric functions topic was 39.28 with a standard deviation of 13.32, and females scored higher than males. Further analysis using an independent samples *t*-test showed that *t* = 1.106 and *p* = 0.14 > 0.05, indicating no significant difference in scores between male and female students. According to the new structural method, students’ mean structural score for the trigonometric functions topic was 46.35 with a standard deviation of 18.42, and females scored higher than males. Further analysis using an independent samples *t*-test showed that *t* = −0.75 and *p* = 0.57 > 0.05, indicating no significant difference in scores between male and female students.

#### 3.2.2 Specific performance of the structural dimension

First, key structural indicators for the two content topics were analyzed using Novak’s classical structural Scoring method (see Supplementary Tables 11–16).

For the sequences functions:

1) Propositions: The number of propositions written by students ranged from 2 to 12. As the number of propositions increased, the number of students varied: the number of students increased from 2 to 6 propositions and decreased from 6 to 12 propositions. The maximum number of students (48) occurred with 6 propositions.
2) Hierarchy: The levels in students’ cognitive structures ranged from 2 to 5. The most common level was 3 (87 students), and 8 students reached 5 levels.
3) Cross-links: 159 students had no cross-links (86.41%), while 25 students had cross-links (13.59%), indicating that most students did not create cross-links for sequences.

For the trigonometric functions:

1) Propositions: The number of propositions ranged from 1 to 9. The number of students increased from 1 to 4 propositions and decreased from 4 to 9 propositions. The maximum number of students (50) occurred with 4 propositions.
2) Hierarchy: Levels ranged from 2 to 5, with the most common being 3 levels (99 students), and 8 students reaching 5 levels.
3) Cross-links: 167 students had no cross-links (90.76%), while 17 students had cross-links (9.24%). Most students did not create cross-links for trigonometric functions.

Second, key structural indicators of students’ sequences and trigonometric functions concept maps were analyzed using the new structural method, focusing on spoke-like and linear structures (see Supplementary Table 17).

For the sequences topic: All students reached the first layer of the spoke-like structure. The second layer was reached by 153 students (83.15%), and the third layer by 87 students (47.28%). A linear structure appeared in 5 students (2.72%).

For the trigonometric functions topic: All students reached the first layer of the spoke-like structure. The second layer was reached by 145 students (78.8%), and the third layer by 73 students (39.67%). A linear structure appeared in 6 students (3.26%).

### 3.3 Cognitive structure performance of Grade 12 students with different levels of mathematical computational ability

This study classifies participants into different levels of computational ability based on their individual test scores relative to the overall mean (M) and standard deviation (SD). Participants with individual scores ≥ M + SD are classified as the “high-ability group”; those with scores within M ± SD are classified as the “medium-ability group”; and those with scores ≤ M − SD are classified as the “low-ability group,” as shown in Table 7. The overall mean score of mathematical computational ability is 490.61, with a standard deviation of 96.44.

**Table 7:** Number and proportion of students in different mathematical computational ability groups.

Group	Secondary dimension	Mean	Proportion
High-ability group	≥587.05	34	18.48%
Medium-ability group	394.16–587.05	95	51.63%
Low-Ability Group	≤394.16	55	29.89%

Notably, the *M* ± SD-based grouping represents a natural stratification approach that may enhance between-group variability.

#### 3.3.1 Correlation analysis between mathematical computational ability and cognitive structure among Grade 12 students

To examine the relationships among key variables, Pearson correlation analysis was conducted. The results (Table 8) showed that mathematical computational ability scores were significantly and positively correlated with scores obtained from the total proposition scoring method, the Novak’s structural scoring method, and the new structural scoring method (all *p* < 0.05), indicating a consistent positive relationship between computational ability and various indicators of cognitive structure.

**Table 8:** Correlation analysis.

Variables	1	2	3	4
Mathematical computational ability	1			
Total proposition scoring method score	0.23^*^	1		
Novak’s classical structural scoring method score	0.76^**^		1	
New structural scoring method score	0.74^**^			1

^**^p < 0.01, ^*^p < 0.05.

#### 3.3.2 Cognitive structure content performance in sequences among Grade 12 students with different levels of mathematical computational ability

**Figure 3:**
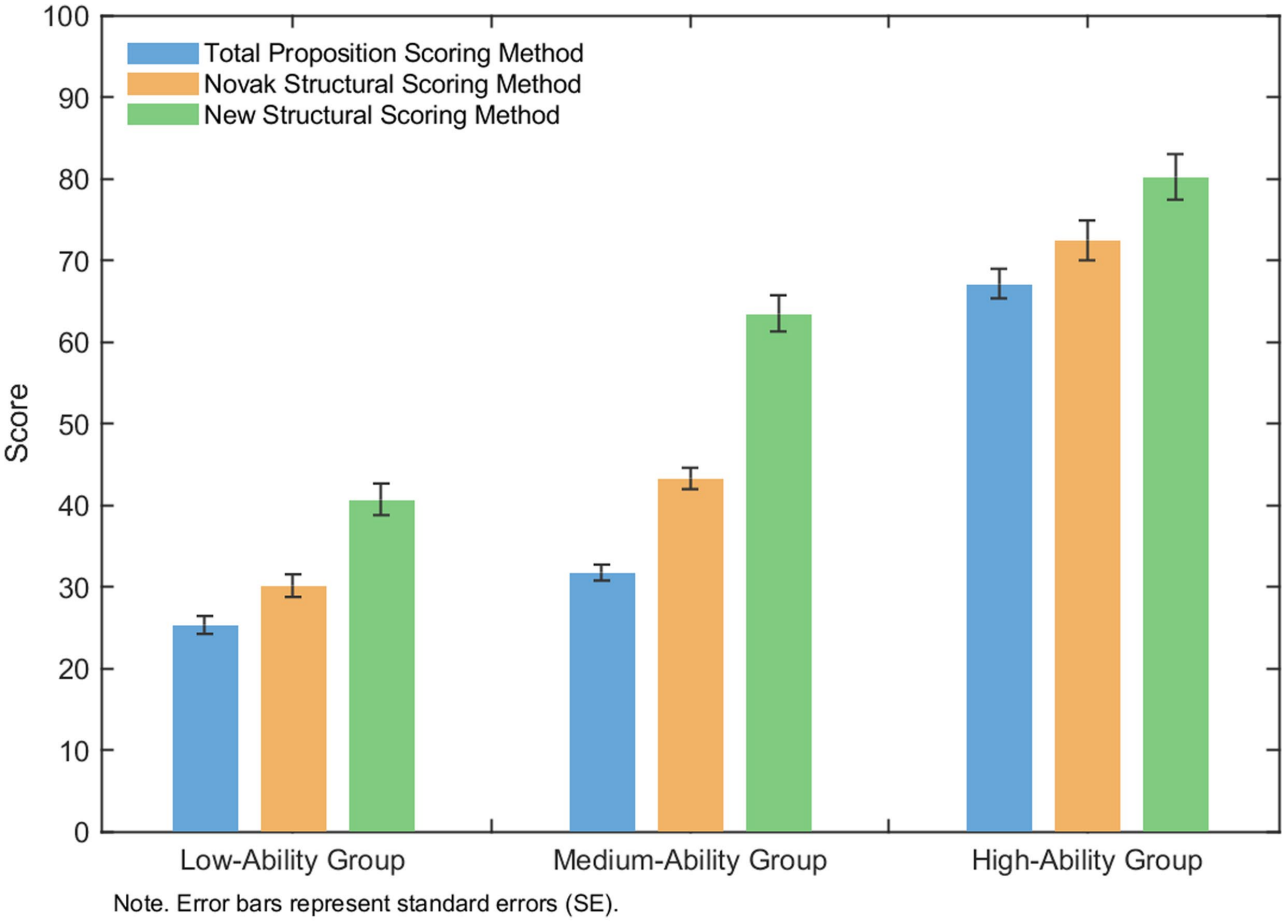
A bar chart comparing scores of low-, medium-, and high-ability groups across three concept map scoring methods: Total Proposition, Novak Structural, and New Structural. Scores increase with ability level, and the New Structural method consistently yields higher scores across all groups. Error bars indicate standard errors.

As shown in Figure 3 and Table 9, among the three assessment methods of sequence cognitive structure, Grade 12 students with high, medium, and low levels of mathematical computational ability exhibited a generally decreasing trend in their scores, with a progressively decreasing trend from the high-ability group to the medium-ability group and then to the low-ability group. One-way ANOVA revealed significant group effects (*p* < 0.001). *Post hoc* comparisons further confirmed that all pairwise differences between groups were statistically significant. Specifically, students in the high-ability group outperformed those in the medium-ability group, and students in the medium-ability group outperformed those in the low-ability group in sequence cognitive structure scores. The effect size results (Cohen’s *d* = 0.72–4.40) suggest that these differences are not only statistically significant but also practically meaningful, with the most pronounced difference observed between the high-ability and low-ability groups.

**Table 9:** Post hoc pairwise comparisons of mathematical computational ability groups for sequence functions.

Measure	Comparison	Mean difference	*p*	Cohen’s *d*
Total proposition scoring method score	High vs. Medium	35.41	<0.001	3.50
	Medium vs. Low	6.42	<0.001	0.72
	High vs. Low	41.83	<0.001	4.40
Novak’s classical structure scoring method score	High vs. Medium	29.17	<0.001	2.15
	Medium vs. Low	13.13	<0.001	1.10
	High vs. Low	42.30	<0.001	2.80
New structure scoring method score	High vs. Medium	16.72	<0.001	0.85
	Medium vs. Low	22.78	<0.001	1.15
	High vs. Low	39.50	<0.001	2.50

#### 3.3.3 Cognitive structure content performance in trigonometric functions among Grade 12 students with different levels of mathematical computational ability

**Figure 4:**
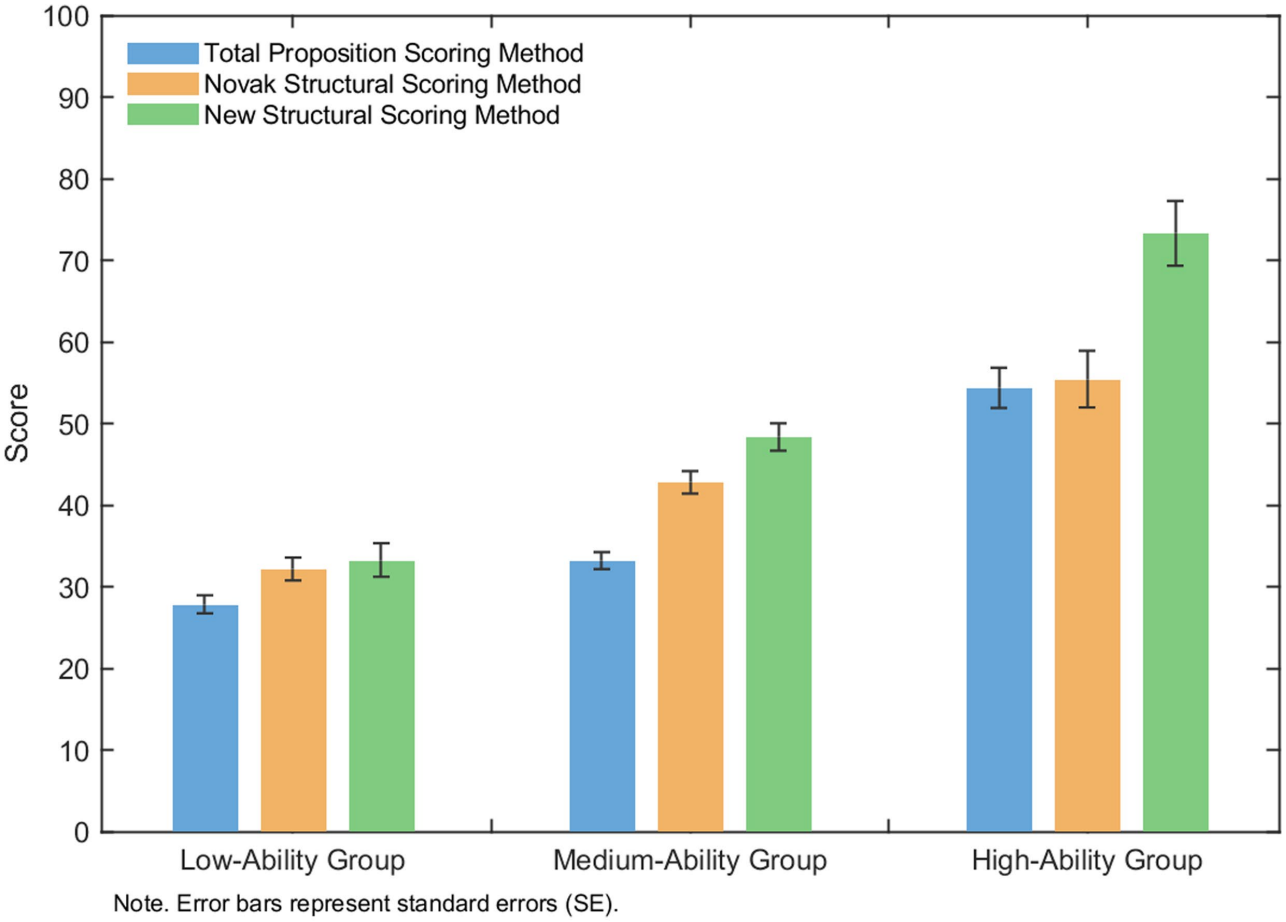
A bar chart showing differences among three scoring methods-Total Proposition, Novak Structural, and New Structural-across low-, medium-, and high-ability groups. Scores increase with both ability level and scoring method, with the New Structural method yielding the highest values. Error bars represent standard errors.

As shown in Figure 4 and Table 10, among the three assessment methods of trigonometric cognitive structure, Grade 12 students with high, medium, and low levels of mathematical computational ability exhibited a generally decreasing trend in their scores, with a progressively decreasing trend from the high-ability group to the medium-ability group and then to the low-ability group. One-way ANOVA revealed significant group effects (*p* < 0.001). Post hoc comparisons further confirmed that all pairwise differences between groups were statistically significant. Specifically, students in the high-ability group outperformed those in the medium-ability group, and students in the medium-ability group outperformed those in the low-ability group in trigonometric cognitive structure scores. The effect sizes (Cohen’s *d* = 0.58–2.20) suggest that these differences are not only statistically significant but also practically meaningful, with the most pronounced differences observed between the high- and low-ability groups.

**Table 10:** Post hoc pairwise comparisons of mathematical computational ability groups for trigonometric functions.

Measure	Comparison	Mean difference	*p*	Cohen’s *d*
Total proposition scoring method score	High vs. Medium	21.17	<0.001	1.70
	Medium vs. Low	5.35	<0.001	0.58
	High vs. Low	26.52	<0.001	2.20
Novak’s classical structure scoring method score	High vs. Medium	12.65	<0.001	0.74
	Medium vs. Low	10.61	<0.001	0.88
	High vs. Low	23.26	<0.001	1.40
New structure scoring method score	High vs. Medium	24.95	<0.001	1.25
	Medium vs. Low	15.07	<0.001	0.95
	High vs. Low	40.02	<0.001	2.10

## 4 Discussion

The hierarchical levels in a concept map indicate the level of knowledge classification of the mapper; the greater the number of levels, the deeper the understanding of the domain (Kinchin et al., 2019). The number of cross-links or horizontal connections in a concept map reflects the mapper’s integrated understanding of knowledge within the domain (Prasetya et al., 2022). This study found that high school students’ mathematical cognitive structures are mainly concentrated between two and five levels. Most students did not form cross-links, and very few exhibited linear structures. This suggests that most students have a relatively solid grasp of basic knowledge, but show insufficient mastery of the relational structure among concepts and propositions, and have not developed systematic and comprehensive mathematical cognitive structures. On the one hand, this may be due to the complexity of high school mathematics content, which makes it difficult for students to establish multiple connections among learning contents in their minds. On the other hand, mathematics teachers tend to emphasize procedural knowledge and problem-solving skills in daily instruction, while neglecting the construction of relationships among concepts, resulting in relatively simple cognitive structures formed by students (Kinchin et al., 2019; Evans and Jeong, 2023). Based on a detailed analysis of the content dimension, the study found that students show significant differences in their understanding of key concepts and methods in the topics of sequences and trigonometric functions. Consistent with previous research, students’ cognitive structures are not only related to learning approaches, but also associated with factors such as individual cognitive abilities; different students thus form different cognitive structures (Luchini et al., 2024).

This study reveals significant gender differences in content scores of mathematical cognitive structures. Female students demonstrated significantly higher content scores in specific thematic cognitive structures compared to male students, though the effect size was relatively small. No significant gender differences were observed in structural scores of mathematical cognitive structures. Female students typically exhibit superior performance in classroom attention, language processing, and learning engagement, which facilitates conceptual knowledge acquisition and expression (Zhang et al., 2024). This may explain the higher content scores observed in females. However, gender differences in mathematical cognitive performance often operate through mediating variables rather than directly influencing knowledge structures (Alonso-Tapia and Ruiz-Díaz, 2022), resulting in smaller effect sizes in content scores and no significant structural differences. Our findings align with existing research indicating high similarity in core mechanisms of mathematical cognitive processing between genders. While gender differences may exist in specific cognitive performance aspects, they generally remain relatively weak (Boada and Cirino, 2023; Zhang et al., 2024).

This study revealed that high-ability high school students scored higher in cognitive structure assessments than middle-ability students, while middle-ability students outperformed low-ability peers, with all differences demonstrating statistical significance and large effect sizes across ability groups. These findings align with existing research confirming distinct mathematical ability stratification among students, where cognitive processing patterns vary significantly across proficiency levels (Xu and Qi, 2022). High-ability students exhibit superior performance in procedural skills, strategy selection, and flexibility—capabilities that facilitate complex knowledge structure formation (Keleş and Yazgan, 2025). Additionally, math-competent students typically demonstrate stronger contextual understanding and superior information integration skills in complex tasks (Sigus and Mädamürk, 2024). The observed disparities in mathematical ability are primarily attributed to sociocultural backgrounds, learning environments, and individual experiences rather than gender factors (Hölscher, 2026).

## 5 Limitations and recommendations

These findings provide certain implications for how mathematics teaching can be used to promote the development of students’ cognitive structures. First, attention should be paid to individual differences in students’ mathematical cognitive structures. Teachers should target specific students and begin with tasks aligned with their zone of proximal development, guiding them to cognitively compare, organize, and reconstruct new and prior knowledge. Second, it is necessary to promote the development of key indicators of students’ mathematical cognitive structures. In the teaching process, mathematics teachers should first improve students’ accurate understanding of concepts; second, guide students to connect new knowledge with prior knowledge; and finally, strengthen the connections among mathematical knowledge to help students construct their own mathematical cognitive structures. During mathematical computation, students should be able to quickly retrieve relevant knowledge and methods from their cognitive structures and identify key entry points for problem-solving.

This study also has some limitations. First, the participants in this study were drawn from Beijing and Shandong Province in China, where educational levels are relatively high, so the findings may provide reference for mathematics education in some developed regions but cannot be fully generalized. Second, the study identified the characteristics of high school students’ mathematical cognitive structures but did not explore the underlying reasons for these phenomena in depth. Finally, this study used correlational analysis and was unable to reveal causal relationships. In the future, the range of research subjects can be expanded to compare the mathematical cognitive structures of students from different regions, and further explore the reasons for differences in mathematical cognitive structures among different groups of students, including curriculum factors, teacher factors, and student factors.

## 6 Conclusion

This study analyzed the mathematical cognitive structures of Grade 12 Students in Beijing and Shandong, China, using three concept map scoring methods. The findings show that the hierarchical levels of students’ mathematical cognitive structures are mainly concentrated between two and five levels. Most students did not form cross-links, and very few exhibited linear structures. There are significant differences in their understanding of key concepts and methods. Female students scored slightly higher than male students in mathematical cognitive structure, and the scores are positively correlated with mathematical computational ability, showing a pattern of high-ability group > medium-ability group > low-ability group.

## Data Availability

The original contributions presented in the study are included in the article/Supplementary material, further inquiries can be directed to the corresponding author.
