# Supplementary material for: Mathematical cognitive structures in Grade 12 students: a mixed-methods concept map study of gender differences and computational ability
Source: Front Psychol. 2026 May 15;17:1844476. doi: 10.3389/fpsyg.2026.1844476 (PMC13219271; doi:10.3389/fpsyg.2026.1844476)
Supplement: Supplementary file 1 [file Data_Sheet_1.DOCX]

**Supplementary Materials:**

# Test Items for Grade 12 students’ Mathematical Computational Ability (Partial)

1.The domain of$y=\lg(x+1)+\sqrt{3-x}$ is .

2.Given vectors $\vec{a}=(2,3),$ $\vec{b}=(-4,x)$.if $\vec{a}//$,then_____.

3.In a circle :$x^{2}+y^{2}-2x-6y=0$，the longest chord and the shortest chord passing through point$E\text{（0，1）}$ are$AC$and $BD$,respectively.Then the area of the quadrilateral $ABCD$ is ___**___** .

4.Define a binary operation:$a\otimes b=\left\{\begin{matrix} a,a\leq b \\ b,a>b \end{matrix}\right.$，For example,12=1，32=2，Then the maximum value of the function:$f(x)=\sin x\otimes\cos x$is .

5. A sequence $\text{\{}a_{n}\}$ is given with general term $a_{n}=kn+b(\text{where k,b are constants , }k\neq0,n\in N^{*})$，and the sum of its first n terms is:$S_{n}=n^{2}+(\sqrt{2}+1)n+a(n\in N^{*},a$ $\text{is a constant})$

(1)Find the general term of the sequence and the value of $a$

(2)Determine whether there exist three terms ($a_{m}\text{、}a_{p}\text{、}a_{q}(m,p,q\in N^{*},and$ $m<p<q)$) in the sequence that can form a geometric progression. If so, find these three terms; if not, explain why.

6.In triangle $ABC$, the sides opposite angles$A$,$B$ and $C$ are $a$, $b$, and $c$, respectively, satisfying:$\sqrt{3}(\sin^{2}A+\sin^{2}B-\sin^{2}C)=$ $2\sin A\sin B\sin C$.

(1)Find angle $c$；

(2)if $a\cos B+b\cos A=4\sqrt{3}$，find the maximum value of the area of the triangle $ABC$.

7.A bridge engineer plans to construct a catenary arch bridge. The curve of the bridge satisfies a function similar to a catenary:$f(x)=ae^{x}\text{+}be^{-x}$(where$a,b$ are nonzero constants,$e=2.71828\cdots$).


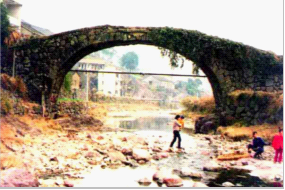

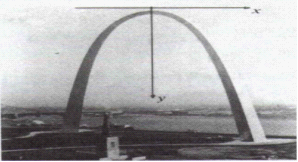


**Figure 1.**

(1)Help the engineer analyze the conditions that $a$ and $b$ must satisfy.

(2)If the function:$f(x)=ae^{x}\text{+}be^{-x}$ (where $a,b$are nonzero constants,$e=2.71828\cdots$)is a monotonic function on$R$,provide one set of values for $a$ and $b$ that satisfies the condition, and explain your reasoning.

# Quality Analysis of the Mathematical Computational Ability Assessment Instrument

(1) Test Difficulty

First, the difficulty of each test item was calculated to obtain the overall distribution of item difficulty for the test.

**Table 1.** Difficulty Distribution of Mathematics Computational Ability Test Items

| **Difficulty** | | **Frequency** | **Percentage (%)** | **Valid Percentage (%)** | **Cumulative Percentage (%)** |
| --- | --- | --- | --- | --- | --- |
| Valid | 0-0.1 | 2 | 11 | 11 | 11 |
|  | 0.1-0.2 | 4 | 21 | 21 | 32 |
|  | 0.2-0.3 | 3 | 16 | 16 | 48 |
|  | 0.3-0.4 | 3 | 16 | 16 | 64 |
|  | 0.4-0.5 | 1 | 5 | 5 | 69 |
|  | 0.5-0.6 | 2 | 11 | 11 | 80 |
|  | 0.6-0.7 | 1 | 5 | 5 | 85 |
|  | 0.7-0.8 | 1 | 5 | 5 | 90 |
|  | 0.8-0.9 | 1 | 5 | 5 | 95 |
|  | 0.9-1.0 | 1 | 5 | 5 | 100 |
|  | 合计 | 19 | 100 | 100 |  |

The item difficulty indices are primarily distributed between 0.2 and 0.6, and several items exhibit satisfactory discriminative power. Overall, the test demonstrates an appropriate level of difficulty.

1. Item Fit Statistics

The MNSQ (weighted fit) values for each item, obtained using the unidimensional Rasch model, are presented in Table 2.

**Table 2.** MNSQ values of each item in the test

| Item Number | MNSQ |
| --- | --- |
| 1 | 1.01 |
| 2 | 0.93 |
| 3 | 0.95 |
| 4 | 0.99 |
| 5 | 1.06 |
| 6 | 0.88 |
| 7 | 0.94 |
| 8 | 0.97 |
| 9 | 1.15 |
| 10 | 0.95 |
| 11 | 1.02 |
| 12 | 0.95 |
| 13 | 0.97 |
| 14 | 0.93 |
| 15 | 1.07 |
| 16 | 1.08 |
| 17 | 1.13 |
| 18 | 1.32 |
| 19 | 1.25 |

All item indices are within the acceptable range of 0.8 to 1.2. Subsequently, item difficulty, fit statistics, and discrimination indices were computed(see Table 3).

**Table 3.** Item fit statistics of the test

| Item Number | Difficulty | Fit (MNSQ) | Discrimination |
| --- | --- | --- | --- |
| 1 | 1.807 | 1.01 | 0.45 |
| 2 | -2.450 | 0.93 | 0.44 |
| 3 | -1.315 | 0.95 | 0.53 |
| 4 | -3.555 | 0.99 | 0.30 |
| 5 | 1.069 | 1.06 | 0.43 |
| 6 | 0.266 | 0.88 | 0.58 |
| 7 | 0.090 | 0.94 | 0.52 |
| 8 | 0.348 | 0.97 | 0.50 |
| 9 | -0.714 | 1.15 | 0.36 |
| 10 | 0.645 | 0.95 | 0.49 |
| 11 | 0.654 | 1.02 | 0.43 |
| 12 | 3.227 | 0.95 | 0.28 |
| 13 | 0.679 | 0.97 | 0.46 |
| 14 | -0.385 | 0.93 | 0.56 |
| 15 | 1.310 | 1.07 | 0.46 |
| 16 | 0.862 | 1.08 | 0.51 |
| 17 | 1.320 | 1.13 | 0.53 |
| 18 | 0.856 | 1.32 | 0.55 |
| 19 | 1.039 | 1.25 | 0.63 |

(3)Wright Map of the Test Items

Using the Rasch model for quantitative analysis, the corresponding Wright map can be obtained. In the figure, the values on the far left represent the logit scale of both person ability and item difficulty. In the middle panel, each “X” represents a certain number of respondents, and the levels of respondents increase from bottom to top. The numbers on the right indicate the item codes.

Respondents and item codes located on the same horizontal line correspond to each other; it is generally interpreted that these respondents have reached the ability level required by the items on that line.

Overall Wright Map of the Test

----------------------------------------------------

3 | |

| |

| |

| |

|12 |

| |

2 | |

| |

| |

X| |

X|15 17 |

X| |

1 XX|19 |

XXXXX|16 18 |

XXXX|11 13 |

XXXXX|10 |

XXXXXX|8 |

XXXXXXXX|6 |

0 XXXXXXXX|7 |

XXXXX| |

XXXXXXXXXX| |

XXXXXXXX|14 |

XXXXXXXXX| |

XXXXXXXXXX|9 |

XXXXXXX| |

-1 XXXXXXXXX|5 |

XXXXXXXX|3 |

XXXXXXXXX| |

XXXXXXXX| |

XXXXXXX|1 |

XXXXXXX| |

-2 XXXXX| |

XXXX| |

XXXX|2 |

XXXXX| |

XXXX| |

XXX| |

-3 XX| |

XX| |

XX| |

X|4 |

X| |

X| |

-4 X| |

====================================================

Each 'X' represents 12.5 cases

(4) Internal Consistency Reliability

The Cronbach’s alpha coefficient reached 0.857, demonstrating good internal consistency reliability. The test is therefore considered appropriate for evaluating high school students’ mathematical computational ability.

# Interview Analysis of High School Mathematics Teachers and Teaching Researchers

**(1)Interview Participants**

Eight outstanding high school mathematics teaching researchers and teachers from Beijing and Shandong who participated in this study were selected for interviews.

**(2)Interview Outline**

Question 1: In your opinion, how is the importance of mathematical computational ability reflected for high school students?

Question 2: What aspects do you think are included in mathematical computational ability?

Question 3: What factors do you think influence high school students’ mathematical computation?

Question 4: In teaching mathematical computation at the high school level, which aspects of students’ learning do you focus on most?

Question 5: What do you think are effective approaches to promoting mathematics teaching for high school students?

**(3)Interview Analysis**

An inductive analysis of the teacher interviews was conducted:

①Regarding how to cultivate students’ computational ability, each teacher had unique perspectives. Their responses can be summarized into the following five aspects:

1. developing a deep understanding of relevant concepts;
2. consolidating computational experience through practice;

(c) optimizing strategies to guide computational approaches;

(d) strengthening computation through formula memorization and attribution;

(e) emphasizing computational rules and theorems.

②Insufficient computational ability can negatively affect students’ learning. Concerning which aspects of mathematical computational ability should be developed, the interviewed teachers proposed the following six aspects:

(a) understanding fundamental knowledge such as mathematical concepts and computational rules;

(b) understanding and applying computational laws;

(c) cultivating students’ ability to select appropriate computational approaches;

(d) fostering reflection and improvement after computation;

(e) developing awareness of equivalent transformation and self-checking;

(f) enhancing students’ ability in mathematical modeling.

③ Teachers identified the main factors influencing students’ computational ability as the following seven aspects:

(a) mathematical cognitive structure;

(b) comprehension of problem information;

(c) mastery of basic computational theorems;

(d) selection of computational approaches;

(e) modeling ability;

(f) students’ own computational skills;

(g) understanding and memorization of computational rules.

# Analysis of Students’ Performance on Typical Problems(Partial)

1.The domain of$y=\lg(x+1)+\sqrt{3-x}$ is .

**Problem Analysis:**This is a basic-level problem. The key to solving it lies in correctly understanding the mathematical computational objects. If students can correctly identify the conditions under which the logarithmic function and the square root function are defined, and then construct corresponding inequalities to determine the valid ranges and take their intersection, the final answer can be obtained. The overall accuracy rate for this item is relatively high, at 88.47%.

**Based on students’ responses and interview analysis, the types of errors are summarized as follows:**

Error Type 1:Students incorrectly understood the mathematical computational objects. They assumed that the argument of the logarithmic function must be ≥ 0 and that the expression under the square root must also be ≥ 0, resulting in the interval [-1, 3). This error occurs because students do not understand the domain restrictions of logarithmic functions and square root functions, leading to incorrect interpretation of the mathematical computational objects.

Error Type 2:Students incorrectly assumed that the argument of the logarithmic function must be ≥ 0, obtaining the interval [-1, 3]. This reflects a misunderstanding of the domain of the logarithmic function, leading to incorrect interpretation of the mathematical computational objects.

Error Type 3:Students ignored the condition that the expression under the square root must be ≥ 0 and obtained (-1, 3). This error arises from a lack of understanding of the domain restrictions of square root functions, resulting in incorrect interpretation of the mathematical computational objects.

Error Type 4:Students only considered the domain of the logarithmic function and ignored the validity condition of the square root function, obtaining (1, +∞). This reflects a misunderstanding of square root domain conditions, leading to incorrect interpretation of the mathematical computational objects.

Error Type 5:Students only considered the domain of the square root function and ignored the condition for the logarithmic function, obtaining (-∞, 3]. This reflects a misunderstanding of logarithmic domain conditions, leading to incorrect interpretation of the mathematical computational objects.

Error Type 6:Students incorrectly understood the mathematical computational objects and misinterpreted the properties of logarithmic functions and square root functions, resulting in an incorrect solution such :*x<3 ,x≠1*. This error occurs because students do not understand the domain of the logarithmic function’s argument and the valid range of the square root expression, leading to a misunderstanding of the mathematical computational objects and consequently an incorrect answer.

Error Type 7:Students failed to compute correctly. Although they established the correct system of inequalities, they incorrectly solved it and obtained [3, +∞). This type of error is caused by computational mistakes.

2.Given vectors $\vec{a}=(2,3),$ $\vec{b}=(-4,x)$.if $\vec{a}//$,then_____.

**Problem Analysis:**This is a fill-in-the-blank question. The content of this item concerns plane vector problems under the geometry and algebra theme. In terms of mathematical objects, the problem is relatively simple, and students can easily recognize that the object of operation is vectors. The main focus of this item is the coordinate representation of given vectors and their parallel relationship, which requires transforming vector relations into coordinate relationships and reflects students’ mastery of computational rules.To solve this type of problem, students need a clear understanding of how parallel vectors can be expressed in coordinate form. This item mainly assesses students’ level of conceptual understanding. Based on a correct understanding of vectors, students are expected to skillfully convert between vector relationships and their coordinate representations and solve the resulting equations accurately. The overall accuracy rate of this item is relatively high, at 94.5%.

**Based on students’ responses and interview analysis, the types of errors are summarized as follows:**

Error Type 1:Students failed to establish a correct computational strategy and did not know how to transform two parallel vectors into their coordinate representation. This error occurs because students are unable to effectively apply the mathematical idea of transformation, leading to an incorrect answer.

Error Type 2:Students selected an incorrect computational method. Although they understood that parallel vectors need to be transformed into coordinate form, they confused the coordinate representations of vector parallelism and perpendicularity during the computation process. This error arises from students’ failure to master and distinguish between the coordinate transformations of vector parallel and perpendicular relationships, resulting in an incorrect choice of method and thus an incorrect answer.

Error Type 3:Students made procedural errors in computation. After correctly transforming the vector parallel relationship into its coordinate form and establishing the correct equation, they made mistakes in solving the equation, such as incorrect sign changes during transposition. This type of error is likely due to carelessness in computation or insufficient ability in solving equations, leading to an incorrect final answer.

# Case Study of Cognitive Structure Scoring

**Table 4.** Scores Based on the Total Proposition Scoring Method(Yin et al., 2005)

| Structural Indicator | | Weight (points) | Quantity (items) | Score(points) |
| --- | --- | --- | --- | --- |
| Concept | | 1 | 33 | 33 |
| Linking Words | | 1 | 6 | 6 |
| Proposition | Proposition-vague or incorrect | 0 | 8 | 0 |
|  | Proposition-simple or incomplete | 1 | 12 | 12 |
|  | Proposition-accurate and meaningful | 2 | 0 | 0 |
| Total | | 51 | | |

**Table 5.** Scores Based on the Novak’s Classical Structural Scoring Method(Novak et al., 1984)

| Type | Weight (points) | Quantity (items) |  | Score(points) |
| --- | --- | --- | --- | --- |
| Relationship (Proposition) | 1 | 7 |  | 7 |
| Hierarchy | 5 | 4 |  | 20 |
| Cross-links | 10 | 0 |  | 0 |
| Examples | 1 | 25 |  | 25 |
| Total |  | 52 | | |

**Table 6.** Scores Based on the New Structural Scoring Method(Tian, 2008)

| Category | Weight (points) | Quantity (items) | Score(points) |
| --- | --- | --- | --- |
| First-level spoke structure | 1 | 7 | 7 |
| Second-level spoke structure | 2 | 21 | 42 |
| Third-level spoke structure | 3 | 3 | 9 |
| Spoke structure subtotal | 58 | | |
| Line structure | 1 | 0 | 0 |
| Line nodes | 1 | 0 | 0 |
| Leaf nodes | 1 | 25 | 25 |
| Total | 83 | | |

# ****Original Images of Concept Map Examples from Some Students(****Partial****)****


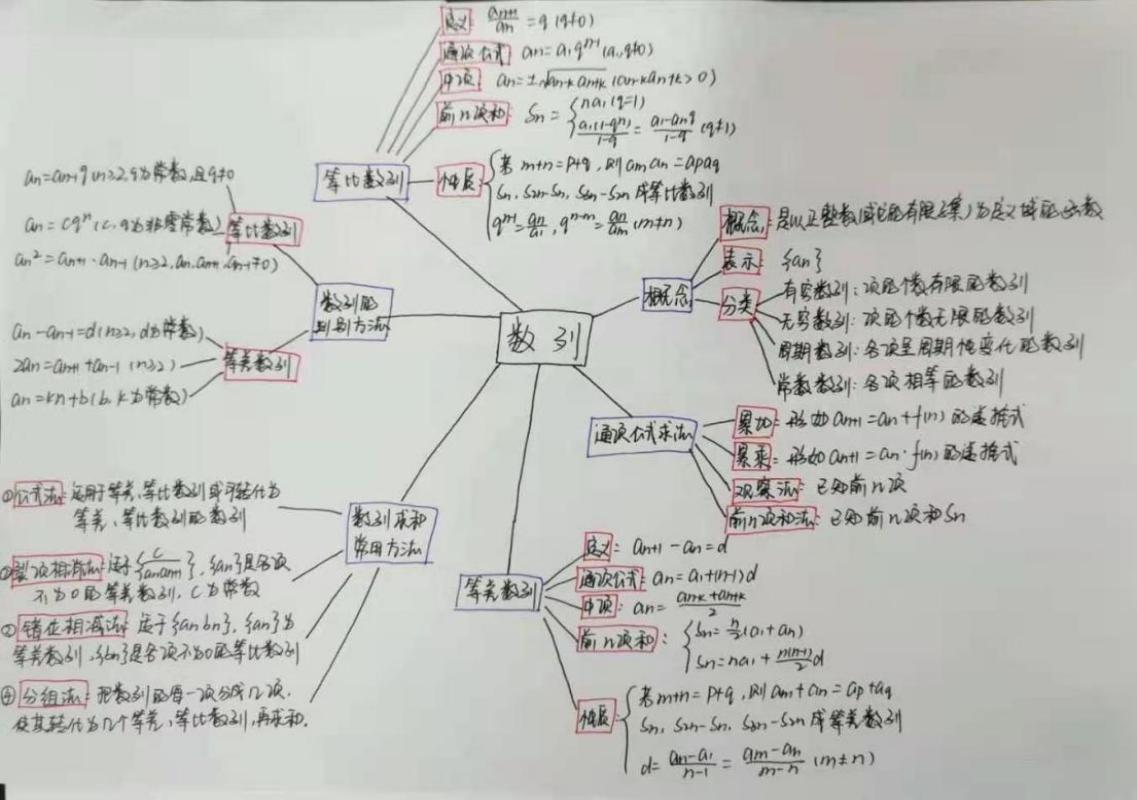


**Figure 2.** Original concept map of sequences


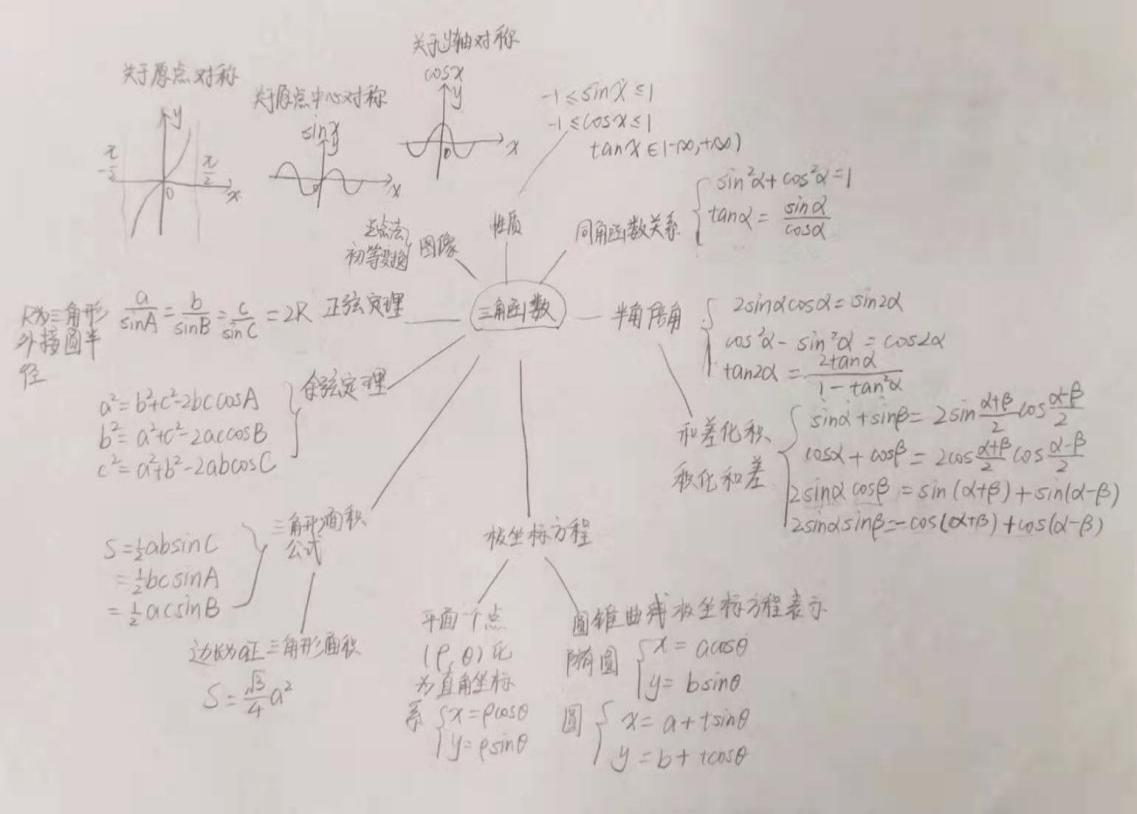


**Figure 3.** original concept map of trigonometric functions


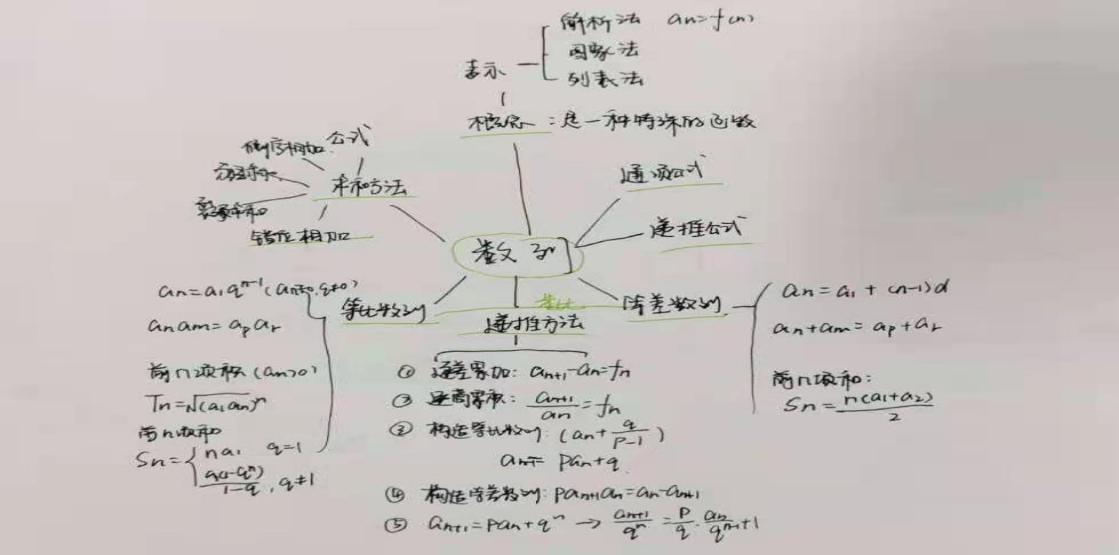


**Figure 4.** Original concept map of sequences


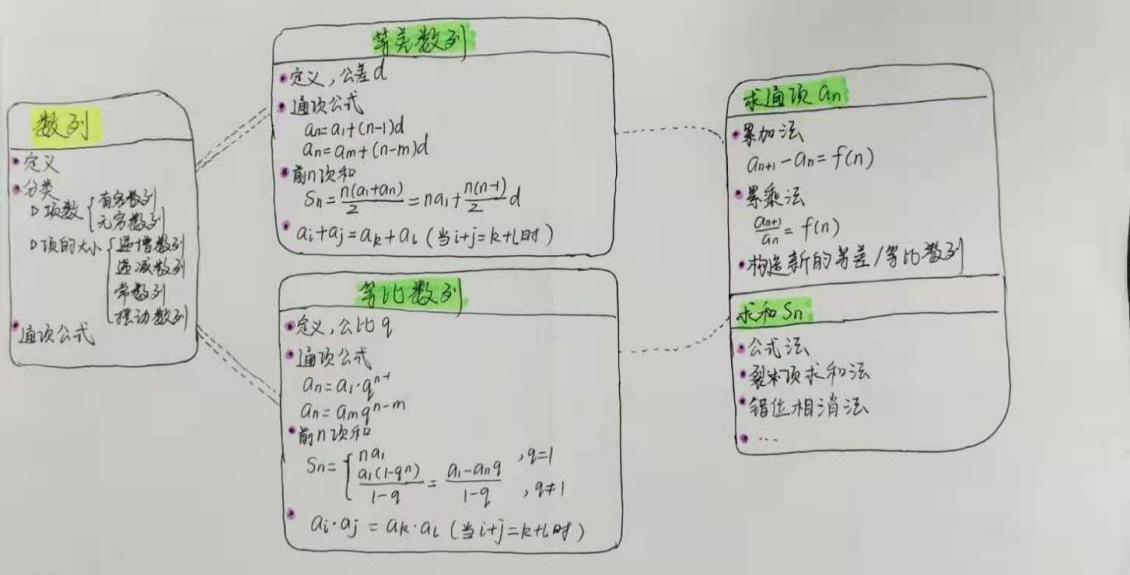


**Figure 5.** Original concept map of sequences

# Results（Table 7-Table 17）

**Table 7.** Frequency and Percentage of Main Basic Concepts and Formulas Presented in the Sequences Functions(Yin et al., 2005)

| Basic Concept or Formula | Frequency | Percentage | Basic Concept or Formula | Frequency | Percentage |
| --- | --- | --- | --- | --- | --- |
| Sum of the first n terms of an arithmetic sequence | 173 | 94.02% | Terms and classification of sequences | 127 | 69.02% |
| General term formula of a geometric sequence | 173 | 94.02% | Geometric mean of a geometric sequence | 104 | 56.52% |
| Definition of an arithmetic sequence | 157 | 85.33% | Representation of sequences | 98 | 53.26% |
| General term formula of an arithmetic sequence | 155 | 84.24% | Properties of arithmetic sequences | 92 | 50.00% |
| Sum of the first n terms of a geometric sequence | 150 | 81.52% | Arithmetic mean of an arithmetic sequence | 92 | 50.00% |
| Definition of a geometric sequence | 148 | 80.43% | Judgment of arithmetic sequences | 58 | 31.52% |
| Definition of a sequence | 132 | 71.74% | Judgment of geometric sequences | 52 | 28.26% |

**Table 8.** Frequency and Percentage of Main Methods Presented in the Sequences Functions(Yin et al., 2005)

| Method Type | Frequency | Percentage | Method Type | Frequency | Percentage |
| --- | --- | --- | --- | --- | --- |
| Misalignment subtraction method | 98 | 53.26% | Recursive representation of arithmetic sequences | 40 | 21.74% |
| Term splitting and cancellation method | 86 | 46.74% | Cumulative addition method | 35 | 19.02% |
| Reverse addition method | 81 | 44.02% | Monotonicity of arithmetic sequences | 35 | 19.02% |
| Group summation method | 75 | 40.76% | Cumulative multiplication method | 29 | 15.76% |
| Recursive formula of sequences | 75 | 40.76% | Sequence construction method | 29 | 15.76% |
| Formula method (direct summation) | 75 | 40.76% |  |  |  |

**Table 9.** Frequency and Percentage of Main Basic Concepts Presented in the Trigonometric Functions (Yin et al., 2005)

| Basic Concept | Frequency | Percentage | Basic Concept | Frequency | Percentage |
| --- | --- | --- | --- | --- | --- |
| Definition of trigonometric functions | 164 | 89.13% | Monotonicity | 82 | 44.57% |
| Graphs | 154 | 83.70% | Symmetry | 60 | 32.61% |
| Periodicity | 146 | 79.35% | *Properties of y*=Asin（ωx+φ） | 55 | 29.89% |
| Domain | 134 | 72.83% | Radian measure | 54 | 29.35% |
| Range | 112 | 60.87% | Circumscribed circle | 12 | 6.52% |
| Even/Odd property | 101 | 54.89% | Inverse trigonometric functions | 8 | 4.35% |

**Table 10.** Frequency and Percentage of Main Formulas and Methods Presented in the Trigonometric Functions (Yin et al., 2005)

| Formula or Method | Frequency | Percentage | Formula or Method | Frequency | Percentage |
| --- | --- | --- | --- | --- | --- |
| Induction formulas (transformation formulas) | 134 | 72.83% | Transformations of trigonometric functions | 61 | 33.15% |
| Sine law | 120 | 65.22% | Area calculation of triangles | 56 | 30.43% |
| Cosine law | 118 | 64.13% | Universal formula (relation formula) | 55 | 29.89% |
| Double-angle formulas | 110 | 59.78% | Sector area formula | 38 | 20.65% |
| Sum-to-product formulas | 95 | 51.63% | Special value method | 28 | 15.21% |

**Table 11.** Distribution of the Number of Propositions in the Sequences Topic Based on the Novak’s Classical Structural Scoring Method(Novak et al., 1984）

| Number of Propositions | 2 | 3 | 4 | 5 | 6 | 7 | 8 | 9 | 10 | 11 | 12 |
| --- | --- | --- | --- | --- | --- | --- | --- | --- | --- | --- | --- |
| Number of Students | 8 | 20 | 24 | 35 | 48 | 32 | 8 | 4 | 2 | 2 | 1 |

**Table 12.** Distribution of Hierarchical Levels in the Sequences Topic Based on the Novak’s Classical Structural Scoring Method(Novak et al., 1984)

| Number of Hierarchical Levels | 2 | 3 | 4 | 5 |
| --- | --- | --- | --- | --- |
| Number of Students | 15 | 87 | 74 | 8 |

**Table 13.** Distribution of Cross-Links in the Sequences Topic Based on the Novak’s Classical Structural Scoring Method(Novak et al., 1984)

| Cross-Links | 0 | 1 | 2 | 3 | 4 |
| --- | --- | --- | --- | --- | --- |
| Number of Students | 159 | 18 | 5 | 1 | 1 |

**Table 14.** Distribution of the Number of Propositions in the Trigonometric Functions Based on the Novak’s Classical Structural Scoring Method(Novak et al., 1984)

| Number of Propositions | 1 | 2 | 3 | 4 | 5 | 6 | 7 | 8 | 9 |
| --- | --- | --- | --- | --- | --- | --- | --- | --- | --- |
| Number of Students | 4 | 15 | 39 | 50 | 41 | 15 | 10 | 6 | 4 |

**Table 15.** Distribution of Hierarchical Levels in the Trigonometric Functions Based on the Novak’s Classical Structural Scoring Method(Novak et al., 1984)

| Number of Hierarchical Levels | 2 | 3 | 4 | 5 |
| --- | --- | --- | --- | --- |
| Number of Students | 34 | 99 | 43 | 8 |

**Table 16.** Distribution of Cross-Links in the Trigonometric Functions Topic Based on the Novak’s Classical Structural Scoring Method(Novak et al., 1984)

| Cross-Links | 0 | 1 | 2 | 3 |
| --- | --- | --- | --- | --- |
| Number of Students | 167 | 11 | 5 | 1 |

**Table 17.** Distribution of Spoke Structures and Chain Structures in the Sequences Functions Based on the new structural scoring method(Tian, 2008)

|  |  | Spoke Structure | | | lineage structure |
| --- | --- | --- | --- | --- | --- |
|  |  | Level 1 | Level 2 | Level 3 |  |
| Sequences Topic | Number | 184 | 153 | 87 | 5 |
|  | % | 100% | 83.15% | 47.28% | 2.72% |
| Trigonometric Functions Topic | Number | 184 | 145 | 73 | 6 |
|  | % | 100% | 78.8% | 39.67% | 3.26% |
